# Editorial: Integrating motivation and attention: behavioral and neural perspectives

**DOI:** 10.3389/fnhum.2025.1729668

**Published:** 2025-11-07

**Authors:** Giampiero Bardella, Emiliano Brunamonti, Suliann Ben Hamed, Fabio Di Bello

**Affiliations:** 1 Department of Physiology and Pharmacology, Sapienza University of Rome, Rome, Italy; 2 Institut des Sciences Cognitives Marc Jeannerod, CNRS, UMR5229, 69675 Bron Cedex, France

**Keywords:** attention, motivation, goal directed behavior, cognitive control, working memory

Motivation refers to a range of urges aimed at meeting a variety of internal (e.g., physiological needs) and external (e.g., social appreciation) demands (Maslow, 1943). While motivation is known to influence cognition broadly, its direct role in the control of attention remains uncertain. For these reasons, understanding how motivational and attentional mechanisms interact to shape complex behavior represents one of the most intriguing topics in cognitive neuroscience. In this editorial, we feature a collection of recent articles addressing such interactions from complementary behavioral and neural perspectives. Together, they provide converging empirical evidence that motivational and attentional mechanisms are not separate regulatory processes. Instead, they appear deeply intertwined, jointly influencing and shaping the cognitive processes underlying goal-directed behaviors (Di Bello et al., 2019; Mogg et al., 2003).

An intriguing aspect about the neurophysiological correlates of this interaction emerges from the work of Narvaria et al. They elucidated how motivational relevance, acquired through repetition, shapes attentional control within visual working memory. By combining behavioral performance with EEG spectral analysis, the authors demonstrated that familiar stimuli, bearing implicit motivational salience from prior exposure, elicit desynchronization in the beta band, which can be associated with efficient retrieval. In contrast, novel items engage frontal theta and parietal alpha rhythms, reflecting sustained cognitive control. These neural signatures reveal how motivation from learned relevance and attention jointly optimizes memory operations. In addition, the findings advance experimental understanding by linking repetition-based facilitation to measurable oscillatory mechanisms. These mechanisms mediate the dynamic interaction of motivation and attention during mnemonic processing.

Another domain in which motivation-attention interference exerts a measurable effect is motivational learning and attentional automaticity guided by social and non-social cueing. In this Research Topic, Salera et al. contributed by investigating how experience-dependent motivational significance transforms transient orienting responses into stable, value-driven attentional habits. The authors demonstrated that only cues with motivational or affective significance, such as gaze direction, generate persistent attentional biases by systematically varying cue predictiveness across social and non-social signals. Habitual attention thus arises from the gradual internalization of motivational contingencies, where learned value guides perceptual priority independently of conscious intention. This idea is coherent with previous research (Chacón-Candia et al., 2023; Salera et al., 2024; Di Bello et al., 2025) showing how neural and behavioral evidence converge to suggest that attention becomes automatic when influenced by learned value, rather than solely by physical salience.

A strong relationship has also been proposed between spatial attention and motor action/inhibition. Haque et al. provided further insights through a non-predictive spatial cueing stop-signal task. They showed that initial exogenous cueing was not beneficial for action stopping. However, after this initial period, inhibition of return (IOR) emerges, decreasing response probability in valid vs. invalid trials. This temporally specific interaction suggests that attention operates as a gating mechanism for inhibitory processes depending on time-dependent motor and perceptual contingencies. This is in contrast with previous studies suggesting that cue predictiveness may play a key role in both preventing initial global stopping and enhancing perceptual processing of the stop signal (Haque et al., 2024; Di Bello et al., 2022). Overall, these findings deepen our understanding of motivation–attention coupling by showing that adaptive motor inhibition also depends on the temporal dynamics of salient stimulus presentation, operating beyond conscious strategic control (Padmala and Pessoa, 2010, Giuffrida et al., 2023).

In his study, Chang examines the relationship between motivation, imagination, and creativity in digital gaming, combining psychological analyses (SEM) with EEG measures. The EEG data reveal increased activity in brain regions associated with creative thinking and emotional processing, including the prefrontal cortex, parietal cortex, occipital lobes, and amygdala. The findings suggest that imagination mediates the relationship between motivation and creative performance, likely encouraging flexible exploration and attentional control during gameplay.

Although much remains to be understood about the mechanisms that govern the interaction between attention and motivation, the articles in this Research Topic reinforce the idea of a tight relationship between the two systems and highlight the potential of leveraging motivational cues to enhance cognitive performance, learning, and creativity in both experimental and applied contexts.
